# Differential functional change in olfactory bulb and olfactory eloquent areas in Parkinson’s disease

**DOI:** 10.1093/braincomms/fcae413

**Published:** 2024-11-16

**Authors:** Yu Luo, Xinyuan Miao, Suraj Rajan, Adrian G Paez, Xinyi Zhou, Liana S Rosenthal, Alexander Pantelyat, Vidyulata Kamath, Jun Hua

**Affiliations:** Division of MR Research, Russell H. Morgan Department of Radiology and Radiological Science, Johns Hopkins University School of Medicine, Baltimore, MD 21205, USA; F.M. Kirby Research Center for Functional Brain Imaging, Kennedy Krieger Institute, Baltimore, MD 21205, USA; Division of MR Research, Russell H. Morgan Department of Radiology and Radiological Science, Johns Hopkins University School of Medicine, Baltimore, MD 21205, USA; F.M. Kirby Research Center for Functional Brain Imaging, Kennedy Krieger Institute, Baltimore, MD 21205, USA; Department of Radiology, Johns Hopkins Hospital, Baltimore, MD 21287, USA; Department of Neurology, Johns Hopkins University School of Medicine, Baltimore, MD 21205, USA; Division of MR Research, Russell H. Morgan Department of Radiology and Radiological Science, Johns Hopkins University School of Medicine, Baltimore, MD 21205, USA; F.M. Kirby Research Center for Functional Brain Imaging, Kennedy Krieger Institute, Baltimore, MD 21205, USA; Division of MR Research, Russell H. Morgan Department of Radiology and Radiological Science, Johns Hopkins University School of Medicine, Baltimore, MD 21205, USA; F.M. Kirby Research Center for Functional Brain Imaging, Kennedy Krieger Institute, Baltimore, MD 21205, USA; Department of Biomedical Engineering, Johns Hopkins University School of Medicine, Baltimore, MD 21205, USA; Department of Neurology, Johns Hopkins University School of Medicine, Baltimore, MD 21205, USA; Department of Neurology, Johns Hopkins University School of Medicine, Baltimore, MD 21205, USA; Department of Psychiatry and Behavioral Sciences, Johns Hopkins University School of Medicine, Baltimore, MD 21205, USA; Division of MR Research, Russell H. Morgan Department of Radiology and Radiological Science, Johns Hopkins University School of Medicine, Baltimore, MD 21205, USA; F.M. Kirby Research Center for Functional Brain Imaging, Kennedy Krieger Institute, Baltimore, MD 21205, USA

**Keywords:** neurodegenerative, sensory, high field MRI, susceptibility artefact, voxel selection

## Abstract

Olfactory dysfunction, or hyposmia, frequently occurs as a prodromal symptom and ongoing sign of Parkinson’s disease. Functional MRI is a powerful tool for studying functional changes in the olfactory brain regions in patients with Parkinson’s disease. However, existing studies show inconsistent results and no study has measured olfactory functional MRI abnormalities in the human olfactory bulb directly. This is mainly due to the well-known susceptibility artefacts in conventional functional MRI images that affect several key olfactory-eloquent brain regions, and especially the olfactory bulb. In this study, olfactory functional MRI was performed using a recently developed functional MRI approach that can minimize susceptibility artefacts and measure robust functional MRI signals in the human olfactory bulb during olfactory stimulation. Experiments were performed on high magnetic field (7 T) in 24 early (<5 years of parkinsonian symptoms) Parkinson’s disease patients and 31 matched healthy controls. Our data showed increased functional MRI signal changes (ΔS/S) in the olfactory bulb in patients with early Parkinson’s disease, which correlated with behavioural olfactory measures. Temporally, functional MRI signals in the olfactory bulb returned to the pre-stimulus state earlier after reaching peak amplitude in patients with early Parkinson’s disease, implicating a faster olfactory habituation effect. The piriform cortex showed reduced numbers of activated voxels in patients with early Parkinson’s disease, which correlated with behavioural olfactory assessment. Several secondary olfactory regions including the orbitofrontal cortex, temporal pole and amygdala exhibited reduced numbers of activated voxels and increased functional MRI signal changes in patients with early Parkinson’s disease. Our data also showed that functional MRI results are highly dependent on voxel selection in the functional analysis. In summary, we demonstrate differential spatial and temporal characteristics of olfactory functional MRI signals between the primary and secondary olfactory regions in patients with early Parkinson’s disease. These results may assist the development of novel quantitative biomarkers (especially in the early stages of Parkinson’s disease) to track and predict disease progression, as well as potential treatment targets for early intervention.

## Teaser

Functional MRI in the olfactory bulb is challenging. Luo *et al*. applied a new MRI approach to measure functional signals in the olfactory bulb directly, and report differential spatial and temporal changes of olfactory functional MRI signals between the primary and secondary olfactory regions in patients with early Parkinson’s disease.

## Graphical Abstract

**Graphical Abstract:**
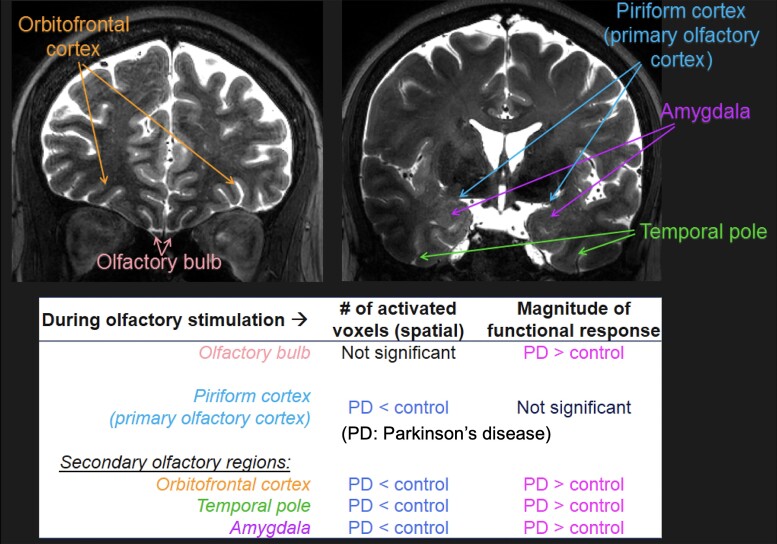

## Introduction

Olfactory dysfunction is both a prodromal feature and an early clinical sign in Parkinson’s disease.^1-4^ Up to 80–90% of sporadic Parkinson’s disease cases show olfactory deficits in the early stage. Olfactory dysfunction, or hyposmia, has been incorporated into the Movement Disorder Society (MDS) clinical diagnostic criteria for Parkinson’s disease.^5,6^ The α-synuclein pathology, which is considered the hallmark pathology of Parkinson’s disease,^7^ often occurs first in the olfactory bulb (OB) and piriform cortex (primary olfactory cortex) before spreading to the brainstem and midbrain regions.^8^ Therefore, olfactory dysfunction may play an important role in the pathogenesis of Parkinson’s disease, and the investigation of functional brain changes underlying olfactory impairment in Parkinson’s disease may aid the development of sensitive and quantitative *in vivo* biomarkers for this disease.

To date, psychophysical testing remains the gold-standard method for assessment of olfaction in humans.^9^ The University of Pennsylvania Smell Identification Test (UPSIT) is the most commonly used measure and involves presenting scratch-and-sniff odours and selecting the correct odour from several listed choices.^10^ The results from this and other behavioural olfactory tests can be subjective and may not accurately reflect the underlying functional changes in different brain regions of the olfactory system. Blood oxygenation level dependent (BOLD) functional MRI is a powerful and versatile tool for studying human brain functions. It has been used to study brain activation in response to olfactory stimulation in Parkinson’s disease patients. Alterations in spatial and temporal characteristics of olfactory fMRI signals from Parkinson’s disease patients compared to controls have been reported in the piriform cortex, which is considered the primary olfactory region in the human brain, as well as in several secondary olfactory regions such as the orbitofrontal cortex, insula, amygdala and hippocampus.^11-14^ Nevertheless, some results from existing studies in Parkinson’s disease patients appear to be contradictive and inconclusive, showing opposite changes in the same regions.^11-14^ Furthermore, to our knowledge, no study has examined olfactory fMRI abnormalities of the OBs in Parkinson’s disease patients. Olfactory information is first projected to the OB from olfactory sensory neurons in the nasal epithelium, and information is then relayed to the piriform cortex and subsequent subcortical and cortical regions.^15^ A major challenge for olfactory fMRI in humans is the well-known susceptibility artefacts from the nearby sinonasal cavity and temporal bone that cause signal dropout and distortion in the conventional gradient echo (GRE) echo-planar-imaging (EPI) based BOLD fMRI images.^16-21^ This affects several key olfactory-eloquent brain regions such as the piriform cortex and orbitofrontal cortex, and the OB.^16-19,21,22^ Due to its small size and close proximity to the nasal cavity, the susceptibility artefacts are usually overwhelming in the human OB, which makes it inaccessible for conventional GRE EPI BOLD fMRI.^23^ We recently demonstrated that the T2-prepared (T2prep) BOLD fMRI^24^ approach can detect robust fMRI signals in the human OB during olfactory fMRI experiments while maintaining whole brain coverage.^25^ The T2prep BOLD fMRI method was originally designed to minimize the susceptibility artefacts in fMRI images caused by metallic head implants,^26^ blood products and/or calcifications in presurgical fMRI.^26,27^ When applying T2prep BOLD fMRI for olfactory fMRI in healthy human subjects, functional activation in the OB and other olfactory-eloquent brain regions can be measured with good intra-subject reproducibility.^25^ T2prep BOLD fMRI has also shown improved sensitivity compared to conventional GRE EPI BOLD fMRI in the OB and olfactory-eloquent brain regions in human subjects.^25^

In this study, olfactory fMRI was performed using T2prep BOLD fMRI in a cohort of early-stage Parkinson’s disease patients and healthy control subjects. MRI scans were performed on high magnetic field (7 T) to take advantage of its enhanced sensitivity. Spatial and temporal characteristics of the fMRI signals measured in the OB and olfactory-eloquent brain regions were investigated and compared between early Parkinson’s disease patients and controls. The relationship between olfactory fMRI measures and behavioural olfactory assessment performance was evaluated.

## Materials and methods

### Study cohort

A total of 55 participants, including 24 early Parkinson’s disease patients and 31 healthy controls were recruited for this study. After initial data screening, nine subjects (two early Parkinson’s disease patients and seven healthy controls) were excluded from final analysis due to incomplete data acquisition or poor image quality (excessive motion or artefacts during MRI). The demographic information for the remaining subjects [22 early Parkinson’s disease patients (11 females, 11 males) and 24 healthy controls (13 females, 11 males)] is summarized in Table 1. Parkinson’s disease patients for this study were recruited from a tertiary referral centre and diagnosed by Movement Disorders Neurologists using the Movement Disorder Society criteria.^6^ Only Parkinson’s disease patients with <5 years of parkinsonian symptoms were included. No study participant had history of neurological or psychiatric disorders other than Parkinson’s disease. Individuals with sinus surgery, craniofacial abnormalities, or nasal trauma or surgery; and individuals who reported respiratory infections, sinus allergies, or symptoms of a common cold within a month prior to the study visit were excluded. All participants were right-handed and non-smokers. According to the Declaration of Helsinki, written informed consent was obtained from all subjects prior to their participation and this study has been approved by the Johns Hopkins Institutional Review Board.

**Table 1:** Demographic information and behavioural assessment

	Early Parkinson’s disease patients	Healthy controls	*P*
*N*	22	24	N/A
Age (year)	65 ± 2^a^	45 ± 3	<0.01*
Sex (female/male)	11/11	13/11	>0.1^b^
Parkinson’s disease duration (year)	3.7 ± 0.4	N/A	N/A
UPSIT total score	19.4 ± 2.1	31.3 ± 0.7	<0.001*

Bold values and * indicate statistically significant results.

^a^Mean ± standard error.

^b^χ^2^ test.

### Behavioural olfactory assessment

The 40-item UPSIT was administrated immediately before MRI to examine odour identification ability in all participants.^10^

### Olfactory stimulation paradigm for functional MRI

An olfactory stimulation setup similar to our prior work^25^ was adopted in the current study. A custom-made multichannel computer-controlled olfactometer (Whiff LLC, Swarthmore, PA, USA)^28^ was placed outside of the scanning room. On one end, the olfactometer was connected to a pressurized air tank to provide constant air flow. On the other end, the olfactometer was connected to Everbilt vinyl tubing (inner diameter: 0.25 inch) that entered the scanning room via a sidewall panel. A nasal cannula (Teleflex, Wayne, PA, USA) was connected to the end of Everbilt vinyl tubing to simultaneously present the odourants to both nostrils of the study participants during MRI. Odourants were stored in separate jars in the olfactometer and delivered using this setup in a constantly flowing humidified air stream (1.5 L per min/nostril) at body temperature with precisely timed pulses. Similar to our previous study,^25^ phenyl ethyl alcohol (PEA, Sigma-Aldrich) was chosen as the stimulant, and odourless mineral oil was used as the control. PEA is known to induce relatively low trigeminal nerve stimulation, and is therefore considered a relatively pure olfactory nerve stimulant.^29-31^ The olfactory stimulation paradigm consisted of a 60-s mineral oil period followed by three blocks of a 60-s PEA period interleaved with a 120-s mineral oil period. The total duration of the paradigm was ∼10 min.

### MRI

All MRI scans were performed on a 7.0 tesla (7 T) Philips MRI scanner (Philips Healthcare, Best, Netherlands) equipped with a 32-channel phased array head coil for signal reception and an eight-channel transmit head coil. Whole brain B0 shimming was performed using the MRCodeTool software (v1.5.9, Tesla DC, Zaltbommel, The Netherlands). Dielectric pads were placed on the sides of the head to improve B1 field homogeneity. A respiratory belt was placed around the abdomen to record the breathing pattern of each participant during fMRI scans.

The MRI scans were carried out according to our previous study.^25^ Briefly, the following MRI scans were performed for each participant with the same order:

1. 3D T_1_-weighted Magnetization-Prepared Rapid Gradient-Echo (MPRAGE) for anatomical imaging: repetition time (TR) = 4500 ms; inversion time (TI) = 563 ms; echo time (TE) = 1.81 ms; voxel = 1 mm isotropic; 180 sagittal slices; total scan duration = 2 min and 15 s.
2. T2prep BOLD fMRI during the olfactory paradigm: TR = 2000 ms; T2prep effective TE = 50 ms; 3D turbo field echo (3D TFE, also known as 3D fast GRE) readout; centric phase encoding profile starting from the centre of k-space (low-high); TR_GRE_/TE_GRE_ = 2.90/1.32 ms; flip angle = 4°; field of view (FOV) = 222 (AP) × 180 (RL) mm^2^; voxel = 1.5 × 1.5 × 1.5 mm^3^; imaging matrix = 148 (AP) × 120 (RL); 84 axial slices covering the entire brain; parallel imaging with SENSE factor = 3 × 3 (RL × FH); partial Fourier (halfscan) = 0.6; total scan duration = 10 min.

### Data analysis

Structural and functional MRI images were processed using the FMRIB Software Library (FSL 6.0.1; FMRIB Oxford, UK), Statistical Parametric Mapping (SPM) software (Version 12, Wellcome Trust Centre for Neuroimaging, London, UK) and in-house programs coded in MATLAB 2018a (MathWorks, Natick, MA, USA).

Data analysis was carried out according to our previous study.^25^ Briefly, the following steps were performed in each data set:

1. Motion correction of all fMRI images was performed using the realignment routine in SPM.
2. Spatial smoothing was performed on all fMRI images using an isotropic Gaussian kernel of 4 mm.
3. Baseline drift in fMRI time series was removed by applying a high-pass filter with a cut-off frequency of 1/180 Hz using FSL.
4. Physiological noise including breathing and cardiac related noises in fMRI data was removed using an independent component analysis (ICA)-based denoising method.^32^
5. Temporal filtering was performed using a low-pass filter with a cut-off frequency of 0.03 Hz.
6. The MPRAGE structural images were co-registered to the T2prep BOLD fMRI images using SPM.
7. All images from each participant were normalized to the Montreal Imaging Institute (MNI) space. The Automated Anatomical Labeling (AAL) atlas^33^ was used to identify the following primary and secondary olfactory regions in the brain: piriform cortex, orbitofrontal cortex, temporal pole, insula, amygdala, hippocampus, parahippocampus, anterior, middle and posterior cingulate cortex. In addition, manual segmentation was performed for the OB similar to our previous work^25^ since it is not included in the AAL atlas. The manual segmentation was performed on all slices by two experienced investigators (X.M. and A.G.P.) independently, after which discrepancies among the investigators were assessed and final selection agreed upon. During the manual segmentation step, all the other regions of interest (ROIs) were visually inspected to ensure acceptable MNI transformation by the two investigators using the same procedures. A total of 11 ROIs were used in subsequent analysis.
8. Functional analysis was performed using the non-parametric Kolmogorov–Smirnov two-sample test^34^ to detect activated voxels in the entire brain during olfactory stimulation (adjusted *P* < 0.01), similar to previous olfactory fMRI studies.^21,22,25^ The number of activated voxels was calculated for each ROI.
9. Relative signal change between PEA and odourless mineral oil (ΔS/S) was calculated for each activated voxel. Similar to previous work,^25^ fMRI signals during the first half of the PEA period and the second half of the mineral oil period were averaged to calculate ΔS/S.
10. Haemodynamic response functions (HRF) were estimated from the fMRI signal time courses averaged over all activated voxels to evaluate the temporal characteristics of olfactory fMRI signal changes. The HRF estimation toolbox software (https://github.com/canlab/CanlabCore/tree/master/CanlabCore/HRF_Est_Toolbox2)^35^ was used to calculate the full width at half maximum (FWHM) and time to peak (TTP) parameters from the HRF. The canonical HRF model with default parameters in the toolbox was used.

Finally, after all data sets were processed, a combined activation map in which all voxels that were significantly activated in healthy controls (similar to our previous study^25^) was generated to represent common voxels that are expected to be activated in healthy subjects. Averaged functional signal changes (ΔS/S) using both individual and combined activation maps were compared between early Parkinson’s disease and control subjects. Note that the HRF calculation was performed using only the individual activation maps.

### Statistics

Statistical analyses were conducted using the Statistical Product and Service Solutions software (SPSS 24.0; Chicago, IL, USA). General linear model was used to compare group differences, with age and ROI volumes measured from structural MRI as covariates. Effect sizes for group differences were estimated using Cohen’s *d*. Linear regression was carried out to evaluate the relationship between fMRI measures and the UPSIT scores. Importantly, age and ROI volumes measured from structural MRI were included as covariates in all analyses including group comparison and linear regression. Multiple comparisons from all ROIs in each analysis were corrected using the false discovery rate (FDR). The Benjamini–Hochberg procedure was performed to control the FDR at *α* = 0.05 for each analysis (group comparisons and linear regression). The same number of ROIs was used to control for FDR in all analysis.

## Results

### Demographic information and behavioural results

The final analytic sample included 22 early Parkinson’s disease patients and 24 healthy control subjects. Their demographic and clinical characteristics are summarized in Table 1. The mean disease duration for early Parkinson’s disease patients was 3.7 ± 0.4 years. Early Parkinson’s disease patients exhibited significantly lower UPSIT scores compared to healthy controls (*P* < 0.001). Significant positive correlation was found between UPSIT scores and disease duration in early Parkinson’s disease patients (*r* = 0.30, *P* = 0.02).

### Olfactory fMRI results

**Figure 1:**
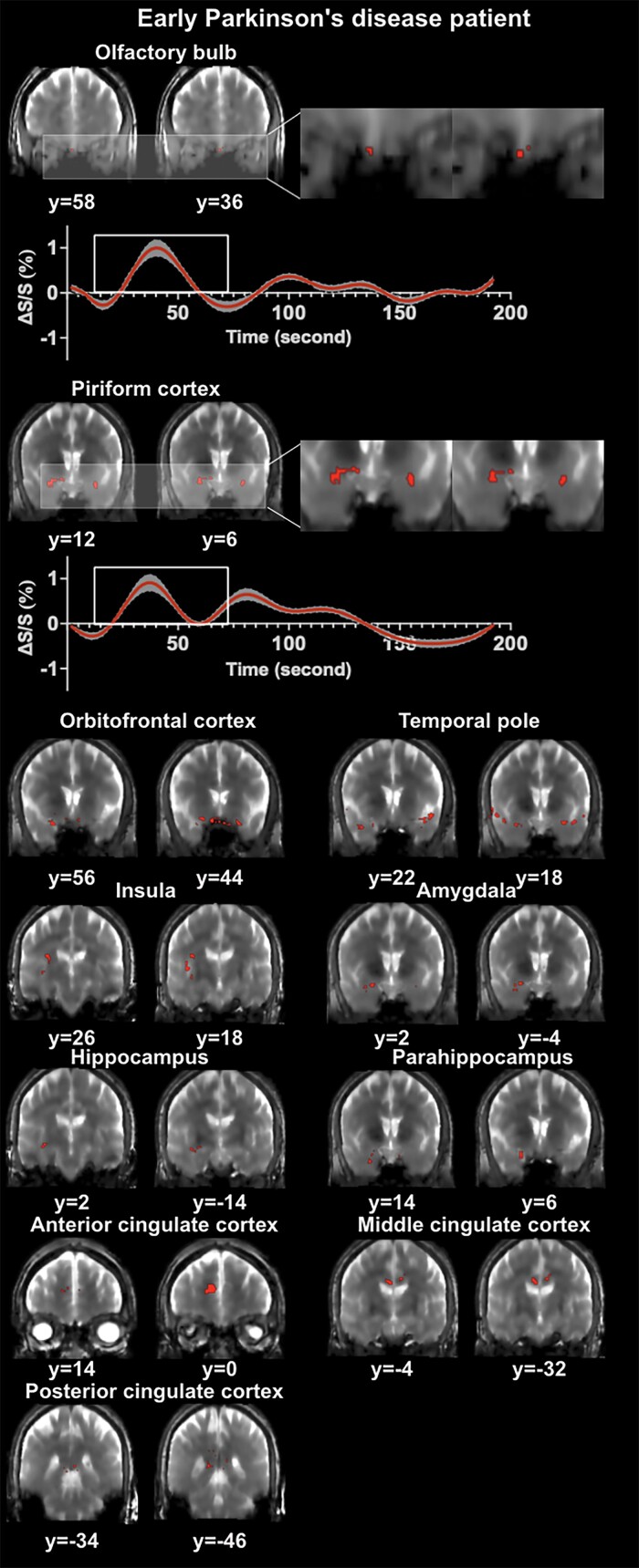
**Representative activation maps and time courses measured by olfactory functional MRI from an early Parkinson’s disease patient are shown.** Activated voxels detected by olfactory functional MRI are marked on the original T2-prepared (T2prep) blood oxygenation level dependent (BOLD) functional MRI images. Two slices in coronal view are shown for each region of interest (ROI). But the entire activated cluster in each ROI covered more slices. Time courses of relative signal changes (ΔS/S) in the olfactory bulb and piriform cortex are shown. The shade indicates the intra-subject standard error. The boxcar marks the olfactory stimulation period. MRI, magnetic resonance imaging.

**Figure 2:**
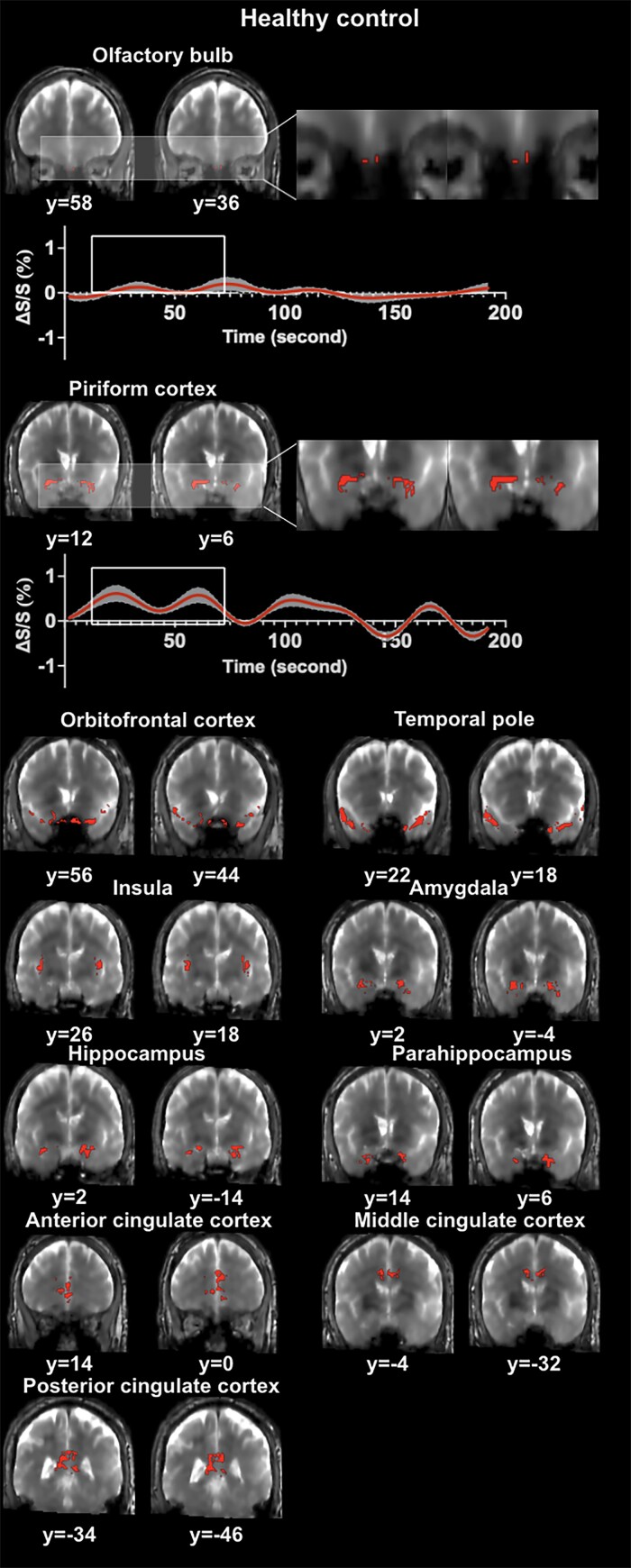
**Representative activation maps and time courses measured by olfactory functional MRI from a healthy control subject are shown.** Activated voxels detected by olfactory functional MRI are marked on the original T2-prepared (T2prep) blood oxygenation level dependent (BOLD) functional MRI images. Two slices in coronal view are shown for each region of interest (ROI). But the entire activated cluster in each ROI covered more slices. Time courses of relative signal changes (ΔS/S) in the olfactory bulb and piriform cortex are shown. The shade indicates the intra-subject standard error. The boxcar marks the olfactory stimulation period. MRI, magnetic resonance imaging.

**Figure 3:**
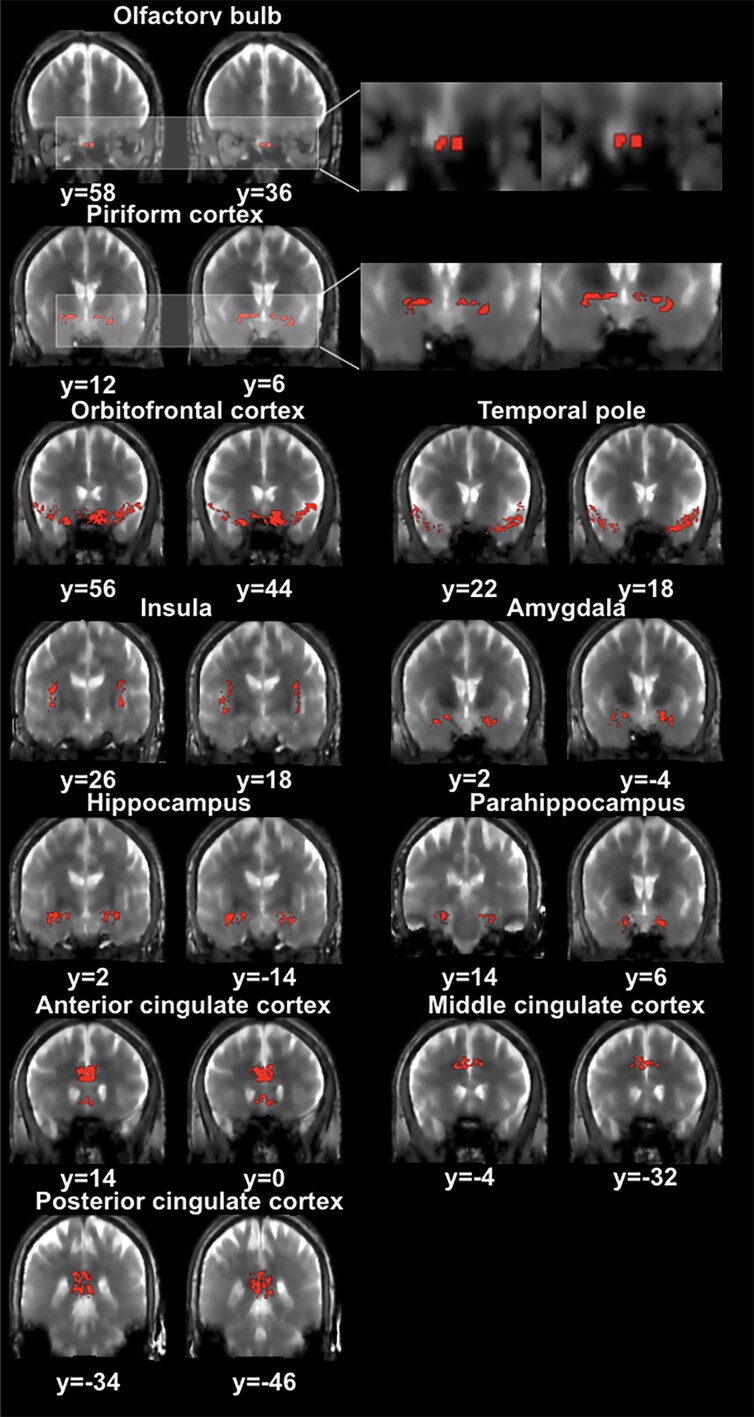
**The combined activation map from healthy controls is shown.** After all data sets were processed, a combined activation map in which all voxels that were significantly activated in healthy controls was generated to represent common voxels that are expected to be activated in healthy subjects.

Figures 1 and 2 show representative activation maps measured by T2prep BOLD fMRI during the olfactory paradigm from an early Parkinson’s disease patient and a healthy control subject, respectively. Figure 3 displays the combined activation map in which all voxels that were significantly activated in healthy controls were included. Most early Parkinson’s disease patients showed less activations in all regions investigated compared to healthy control subjects.

Tables 2–4 summarize the quantitative fMRI results from all subjects. The numbers of activated voxels (Table 2) were significantly reduced in early Parkinson’s disease patients compared to controls in the piriform cortex, as well as the orbitofrontal cortex, temporal pole and amygdala. There was also a decreasing trend for the number of activated voxels in the hippocampus and middle cingulate cortex in early Parkinson’s disease patients compared to controls. Linear regression analysis revealed that lower UPSIT scores, indicative of poorer olfactory function, were significantly associated with fewer numbers of activated voxels in the piriform cortex, temporal pole and orbitofrontal cortex. Interestingly, the number of activated voxels in the OB appeared to be comparable between early Parkinson’s disease and controls.

**Table 2:** Number of activated voxels in each brain region measured by olfactory fMRI

	Early Parkinson’s disease patients (*n* = 22)	Healthy controls (*n* = 24)	Parkinson’s disease versus control			Correlation with UPSIT	
	Voxel number	Voxel number	Relative change (%)^a^	*P*	Effect size^b^	*r*	*P*
Olfactory bulb	28 ± 5^c^	32 ± 5	−12.5	0.19	0.16	0.00	0.99
Piriform cortex	239 ± 33	370 ± 29	−35.3	<0.01*	0.88	0.30	**0**.05*
Orbitofrontal cortex	5468 ± 473	6453 ± 338	−15.3	**0**.02*	0.50	0.38	**0**.01*
Temporal pole	1720 ± 167	2028 ± 135	−15.2	**0**.04*	0.42	0.33	**0**.03*
Insula	2511 ± 293	2764 ± 189	−9.2	0.19	0.22	0.10	0.51
Amygdala	253 ± 32	315 ± 26	−19.7	**0**.03*	0.45	0.19	0.22
Hippocampus	1158 ± 127	1397 ± 92	−17.1	0.07	0.45	0.19	0.23
Parahippocampus	1374 ± 146	1566 ± 120	−12.3	0.12	0.30	0.17	0.26
Anterior cingulate	1692 ± 211	1878 ± 181	−9.9	0.13	0.20	0.02	0.89
Middle cingulate	2505 ± 292	3077 ± 297	−18.6	0.08	0.40	0.00	1.00
Posterior cingulate	534 ± 81	537 ± 60	−0.5	0.37	0.01	0.08	0.61

Bold values and * indicate statistically significant results.

^a^Relative change is defined as: 100 × (mean voxel numbers in early Parkinson’s disease patients − mean voxel numbers in healthy controls)/(mean voxel number in healthy controls) %.

^b^Effect size is estimated using Cohen’s *d*.

^c^Mean ± standard error.

**Table 3:** Olfactory functional MRI signal changes^c^ averaged over activated voxels in each subject

	Early Parkinson’s disease patients (*n* = 22)	Healthy controls (*n* = 24)	Parkinson’s disease versus control			Correlation with UPSIT	
	ΔS/S (%)	ΔS/S (%)	Relative change (%)^a^	*P*	Effect size^b^	*r*	*P*
Olfactory bulb	1.94 ± 0.80^d^	0.78 ± 0.43	148	0.03*	0.40	−0.42	**<0**.01*
Piriform cortex	0.83 ± 0.19	0.87 ± 0.21	−4	0.36	0.04	−0.17	0.27
Orbitofrontal cortex	1.30 ± 0.24	0.54 ± 0.08	140	**<0**.01*	0.90	−0.46	**<0**.01*
Temporal pole	0.97 ± 0.18	0.50 ± 0.11	94	**<0**.01*	0.66	−0.40	**<0**.01*
Insula	0.74 ± 0.16	0.49 ± 0.08	51	0.09	0.41	−0.31	**0**.04*
Amygdala	1.06 ± 0.35	0.49 ± 0.12	116	**0**.04*	0.48	−0.31	**0**.05*
Hippocampus	0.82 ± 0.22	0.52 ± 0.13	57	0.11	0.36	−0.26	0.09
Parahippocampus	0.87 ± 0.26	0.62 ± 0.15	40	0.16	0.25	−0.27	0.08
Anterior cingulate	0.97 ± 0.18	0.62 ± 0.16	56	0.11	0.42	−0.24	0.12
Middle cingulate	0.61 ± 0.16	0.72 ± 0.18	−15	0.47	0.14	−0.02	0.88
Posterior cingulate	0.81 ± 0.25	0.73 ± 0.20	10	0.35	0.08	−0.06	0.70

Bold values and * indicate statistically significant results.

^a^Relative change is defined as: 100 × (ΔS/S in early Parkinson’s disease patients − ΔS/S in healthy controls)/(ΔS/S in healthy controls) %.

^b^Effect size is estimated using Cohen’s *d*.

^c^Olfactory functional MRI signal change: ΔS/S = 100 × (stimulus-on signals − stimulus-off signals)/stimulus-off signals %.

^d^Mean ± standard error.

**Table 4:** Olfactory functional MRI signal changes^c^ in each brain region averaged over the combined activation map from healthy controls

	Voxel number in the combined activation map	Early Parkinson’s disease patients (*n* = 22)	Healthy controls (*n* = 24)	Parkinson’s disease versus control			Correlation with UPSIT	
		ΔS/S (%)	ΔS/S (%)	Relative change (%)^a^	*P*	Effect size^b^	*r*	*P*
Olfactory bulb	31	−0.03 ± 0.22^d^	−0.16 ± 0.17	−81	0.15	0.14	−0.25	0.10
Piriform cortex	413	−0.09 ± 0.12	0.48 ± 0.18	−119	0.04*	0.80	0.30	**0**.05*
Orbitofrontal cortex	6917	0.03 ± 0.12	0.05 ± 0.06	−40	0.26	0.05	0.01	0.94
Temporal pole	1564	−0.18 ± 0.13	0.05 ± 0.07	−460	**0**.03*	0.51	0.38	**0**.01*
Insula	3165	0.19 ± 0.22	0.16 ± 0.09	19	0.42	0.04	−0.14	0.36
Amygdala	226	−0.20 ± 0.15	0.35 ± 0.13	−157	**0**.04*	0.85	0.31	**0**.05*
Hippocampus	1284	0.05 ± 0.19	0.23 ± 0.11	−78	0.32	0.24	−0.09	0.57
Parahippocampus	898	0.06 ± 0.24	0.34 ± 0.15	−82	0.21	0.30	−0.08	0.61
Anterior cingulate	2154	−0.13 ± 0.20	0.36 ± 0.20	−136	**0**.05*	0.52	0.27	0.08
Middle cingulate	2821	−0.26 ± 0.23	0.25 ± 0.19	−204	0.13	0.51	0.31	**0**.04*
Posterior cingulate	787	−0.20 ± 0.21	0.43 ± 0.23	−147	**0**.05*	0.61	0.28	0.07

Bold values and * indicate statistically significant results.

^a^Relative change is defined as: 100 × (ΔS/S in early Parkinson’s disease patients − ΔS/S in healthy controls)/(ΔS/S in healthy controls) %.

^b^Effect size is estimated using Cohen’s *d*.

^c^Olfactory functional MRI signal change: ΔS/S = 100 × (stimulus-on signals − stimulus-off signals)/stimulus-off signals %.

^d^Mean ± standard error.

The fMRI signal changes (ΔS/S, Table 3) averaged over activated voxels in each subject during olfactory stimulation were significantly greater in early Parkinson’s disease patients compared to controls in the OB, orbitofrontal cortex, temporal pole and amygdala. Additionally, the insula showed an increasing trend of ΔS/S in early Parkinson’s disease patients compared to controls. Linear regression analysis revealed significant negative correlations between ΔS/S and UPSIT in the OB, orbitofrontal cortex, temporal pole, amygdala and insula, and a trend for negative correlation in the hippocampus and parahippocampus. Nevertheless, when averaging ΔS/S over all voxels in the combined activation map obtained from healthy controls (Fig. 3), ΔS/S was lower in early Parkinson’s disease patients compared to controls in nearly all ROIs (Table 4), reaching significance in the piriform cortex, temporal pole, amygdala, anterior and posterior cingulate cortex. Linear regression analysis showed significant positive correlations between ΔS/S and USPIT in the piriform cortex, temporal pole, amygdala and middle cingulate cortex, and a trend for correlation in the anterior and posterior cingulate cortex. Note that since the combined activation map had more voxels (Table 4) that may not have been activated in individual activation maps (Table 2), ΔS/S in some ROIs was not detectable. Therefore, only ΔS/S averaged from individual activation maps was used in subsequent analysis.

Table 5 demonstrates the temporal characteristics of ΔS/S averaged over activated voxels in each subject measured by the HRF and its parameters. Early Parkinson’s disease patients exhibited significantly narrower FWHM of the HRF compared to controls in the OB, amygdala and orbitofrontal cortex. Additionally, there was a trend of narrower FWHM in the piriform cortex in early Parkinson’s disease patients compared to controls. No significant difference was found in TTP between early Parkinson’s disease patients and controls in any ROI.

**Table 5:** Temporal characteristics of olfactory functional MRI signals measured by haemodynamic response functions (HRF)

				Parkinson’s disease versus controls			Correlation with UPSIT	
		Early Parkinson’s disease patients (*n* = 22)	Healthy controls (*n* = 24)	Relative change (%)^a^	*P*	Effect size^b^	*r*	*P*
FWHM (s)^c^	Olfactory bulb	2.31 ± 0.69^d^	3.79 ± 0.29	−39	0.01*	0.86	0.33	0.07
	Piriform cortex	3.75 ± 0.42	4.31 ± 0.21	−12	0.08	0.63	0.16	0.40
	Orbitofrontal cortex	3.56 ± 0.46	4.21 ± 0.21	−15	**0**.05*	0.79	0.19	0.32
	Temporal pole	3.63 ± 0.37	4.17 ± 0.25	−12	0.12	0.56	0.01	0.97
	Insula	4.25 ± 0.38	4.42 ± 0.18	−3	0.35	0.14	−0.11	0.55
	Amygdala	3.31 ± 0.55	4.44 ± 0.16	−25	**<0**.01*	0.98	0.13	0.50
	Hippocampus	4.13 ± 0.46	4.08 ± 0.18	1	0.46	0.04	0.03	0.89
	Parahippocampus	4.13 ± 0.38	3.81 ± 0.17	8	0.18	0.38	0.04	0.82
	Anterior cingulate	3.31 ± 0.31	3.77 ± 0.20	−12	0.12	0.54	0.04	0.85
	Middle cingulate	4.06 ± 0.51	4.38 ± 0.20	−7	0.21	0.34	−0.21	0.17
	Posterior cingulate	3.75 ± 0.50	4.46 ± 0.33	−15	0.13	0.54	0.33	0.07
TTP (s)^c^	Olfactory bulb	4.80 ± 1.76	4.92 ± 0.81	−2	0.40	0.25	0.002	0.99
	Piriform cortex	7.08 ± 0.98	7.46 ± 0.51	−5	0.29	0.15	−0.11	0.56
	Orbitofrontal cortex	7.67 ± 1.14	7.50 ± 0.63	2	0.37	0.05	−0.10	0.59
	Temporal pole	7.33 ± 0.68	7.85 ± 0.68	−7	0.33	0.20	0.02	0.94
	Insula	6.75 ± 1.25	7.15 ± 0.63	−6	0.39	0.15	0.04	0.84
	Amygdala	6.92 ± 1.37	8.15 ± 0.66	−15	0.42	0.47	−0.07	0.70
	Hippocampus	6.83 ± 1.14	7.75 ± 0.48	−12	0.10	0.40	−0.02	0.94
	Parahippocampus	5.50 ± 1.37	8.85 ± 0.63	−38	0.06	1.35	0.14	0.46
	Anterior cingulate	7.75 ± 1.16	8.08 ± 0.51	−4	0.44	0.15	0.02	0.90
	Middle cingulate	8.75 ± 1.60	7.27 ± 0.53	20	0.29	0.54	0.07	0.72
	Posterior cingulate	8.25 ± 1.94	8.25 ± 0.62	0	0.43	0.00	−0.21	0.27

Bold values and * indicate statistically significant results.

^a^Relative change is defined as: 100 × (mean HRF parameters in early Parkinson’s disease patients − mean HRF parameters in healthy controls)/(mean HRF parameters in healthy controls) %. HRF parameters include FWHM and TTP.

^b^Effect size is estimated using Cohen’s *d*.

^c^FWHM, full width at half maximum (s); TTP, time to peak (s).

^d^Mean ± standard error.

To assess effects from the spatial smoothing step in the fMRI analysis, Supplementary Table 1 showed fMRI results with and without spatial smoothing in the OB, which is the smallest ROI in this study. Similar results were obtained.

**Figure 4:**
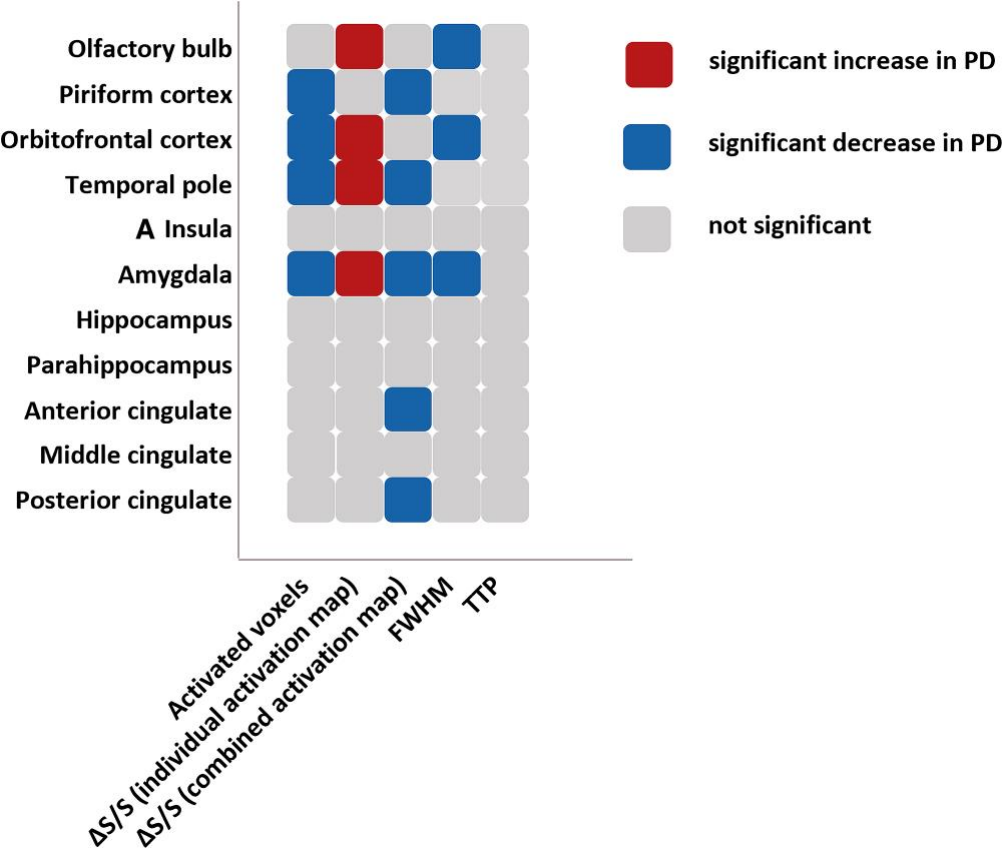
**Summary of the main findings in this study.** Significant results from Tables 2–5 were summarized here for each region of interest (ROI). Changes in number of activated voxels, relative signal changes (ΔS/S) from individual and combined activation maps, the full width at half maximum (FWHM) and time to peak (TTP) measured from the haemodynamic response functions (HRF) in each ROI were summarized. The red boxes indicate that the corresponding measures significantly increased in early Parkinson’s disease patients compared to controls. The blue boxes indicate that the corresponding measures significantly decreased in early Parkinson’s disease patients compared to controls. The grey boxes indicate that the corresponding measures were not significantly different between early Parkinson’s disease patients and controls.

Finally, Fig. 4 summarizes the main findings in our data for each ROI.

## Discussion

In this study, olfactory fMRI was performed to investigate the changes in the spatial and temporal characteristics of fMRI signals in olfactory-eloquent brain regions and the OB in early-stage Parkinson’s disease patients. To the best of our knowledge, this may be one of the first studies showing disease related fMRI changes in the human OB *in vivo*. This was made possible by using T2prep BOLD fMRI, an alternative fMRI approach developed for conducting fMRI in brain regions that are substantially affected by susceptibility artefacts. To date, most fMRI studies in the OB were performed in animals.^36-51^ T2prep BOLD fMRI can detect functional activation in the OB in humans with whole brain coverage. In the OB, our data showed no significant difference between the number of activated voxels in early Parkinson’s disease patients and controls. However, the fMRI signal change (ΔS/S, averaged individually) in the OB was significantly greater in early Parkinson’s disease patients compared to controls, and individuals with greater ΔS/S tend to have lower UPSIT scores on olfactory assessment suggesting negative correlation. This seems to indicate that functional activities in response to olfactory stimulation were more vigorous in the OB of early Parkinson’s disease patients. Temporally, fMRI signal changes in the OB demonstrated a smaller FWHM in early Parkinson’s disease patients compared to controls, but no significant difference in TTP between the two groups. This suggests that fMRI signal in the OB returned to baseline earlier after its peak in early Parkinson’s disease patients compared to controls, implying a faster olfactory habituation effect.

The literature on fMRI signal changes in the OB in Parkinson’s disease patients is very limited. Using electrobulbogram (EBG) responses, Iravani *et al.*^23,52^ reported altered odour-induced EBG responses in Parkinson’s disease patients compared to controls, showing increased or decreased activities in Parkinson’s disease patients in different frequency bands and during different post-stimulus periods. Note that the temporal scales in EBG and fMRI are quite different: whereas EBG recordings are epoched a few seconds before and after the odour presentation, olfactory fMRI is typically performed several ‘minutes’ before, during and after the odour presentation. The underlying mechanisms for olfactory fMRI changes in the OB observed in Parkinson’s disease patients here clearly warrant further investigation using imaging and other techniques.

In contrast to the OB, the piriform cortex, which is considered the primary olfactory cortex in humans, showed significantly lower numbers of activated voxels in early Parkinson’s disease patients, but comparable ΔS/S (averaged individually) between early Parkinson’s disease and controls; individuals with fewer activated voxels in the piriform cortex tended to have lower UPSIT scores (positive correlation). These observations seem to indicate that while fewer neurons were activated in the piriform cortex during olfactory stimulation in early Parkinson’s disease patients, their functional activity levels were comparable between early Parkinson’s disease and controls. This aligns with the majority of existing literature on olfactory fMRI changes in the piriform cortex in Parkinson’s disease patients.^11-14^

Several secondary olfactory regions such as the orbitofrontal cortex, temporal pole and the amygdala exhibited both significantly reduced number of activated voxels and increased ΔS/S (averaged individually) in early Parkinson’s disease patients compared to controls. This may indicate that while there are fewer functioning neurons in these regions in early Parkinson’s disease, the remaining neurons may be exhibiting adaptive compensation through upregulated activity in response to olfactory stimulation.

Interestingly, when averaging over activated voxels in each individual, the functional signal changes (ΔS/S) ‘increased’ in most ROIs in early Parkinson’s disease patients compared to controls, and furthermore showed a negative correlation with UPSIT scores. On the other hand, when averaging over activated voxels in the combined activation map obtained from healthy controls, ΔS/S decreased in most ROIs in early Parkinson’s disease patients, with a positive correlation with UPSIT scores. Note that many voxels in the combined activation map obtained from controls may not be activated in early Parkinson’s disease patients. Thus, ΔS/S from the combined activation map was diluted by these voxels in early Parkinson’s disease patients, leading to an overall decrease in ΔS/S compared to controls. This observation underscores the importance of voxel selection when comparing fMRI signal changes between groups and may at least partially explain the apparent contradictions among existing studies showing either increased or decreased olfactory fMRI signals in these regions in Parkinson’s disease patients.^11-14^

There are several limitations in the current study. First, the early Parkinson’s disease group was significantly older than the control group recruited for this study. Although age was included as a covariate in all statistical analyses in this study, care should be taken when interpreting the results as olfactory dysfunction increases with older age.^53^ A control group that is strictly age-matched with the early Parkinson’s disease group should be used in future studies. The UPSIT scores in the early Parkinson’s disease patients in the current study were lower than typical UPSIT scores in older healthy controls.^31,54,55^ We therefore believe that the results obtained in this study are still meaningful for subsequent studies. Second, only one odour was used in this study. We chose PEA as it induced robust fMRI signals in the OB and other olfactory regions in our previous studies,^25^ and is known to have relatively low trigeminal stimulation.^29-31^ Prior behavioural studies have shown that the olfactory dysfunction in Parkinson’s disease appears to be selective to several specific odours such as gasoline, banana, pineapple, smoke and cinnamon.^56^ Subsequent studies should be designed based on this selective pattern of hyposmia in Parkinson’s disease. Finally, spatial smoothing is commonly used in fMRI studies to improve signal-to-noise ratio (SNR). In this study, we used an isotropic Gaussian kernel of 4 mm for spatial smoothing, same as our previous olfactory fMRI study.^25^ However, such smoothing may introduce additional spatial blurring that is particularly relevant for small brain regions. The same smoothing procedure was performed in early Parkinson’s disease patients and controls to minimize its effect on group comparisons. Nevertheless, the development of more advanced MRI techniques with higher intrinsic SNR should help to alleviate this problem and avoid the use of spatial smoothing in future studies.

In conclusion, we showed that olfactory fMRI can detect significant functional changes in olfactory-eloquent brain regions and OB in early Parkinson’s disease patients. Compared to the conventional GRE EPI-based BOLD fMRI, the T2prep BOLD method used here significantly reduced susceptibility artefacts in several olfactory regions (especially the OB), making it possible to measure functional actives in these regions that may be otherwise inaccessible with conventional fMRI. The spatial and temporal characteristics of the olfactory fMRI signals demonstrated significant regional differences between the primary and secondary olfactory regions. These results may help to improve our understanding of functional brain changes underlying olfactory dysfunction in early Parkinson’s disease. They may also assist the development of novel quantitative biomarkers (especially in early disease stages) to track and predict disease progression, as well as potential treatment targets for early intervention.

## Supplementary Material

fcae413_Supplementary_Data

## Data Availability

Data supporting the conclusions will be made available by the corresponding author upon request via personal communication. In-house programs (code) are included in the supplementary materials.

## References

[1] Montgomery EB Jr, Baker KB, Lyons K, Koller WC. Abnormal performance on the PD test battery by asymptomatic first-degree relatives. Neurology. 1999;52(4):757–762. 10078723 10.1212/wnl.52.4.757

[2] Ponsen MM, Stoffers D, Booij J, van Eck-Smit BLF, Wolters EC, Berendse HW. Idiopathic hyposmia as a preclinical sign of Parkinson’s disease. Ann Neurol. 2004;56(2):173–181. 15293269 10.1002/ana.20160

[3] Ross GW, Petrovitch H, Abbott RD, et al Association of olfactory dysfunction with risk for future Parkinson’s disease. Ann Neurol. 2008;63(2):167–173. 18067173 10.1002/ana.21291

[4] Heinzel S, Berg D, Gasser T, et al Update of the MDS research criteria for prodromal Parkinson’s disease. Mov Disord. 2019;34(10):1464–1470. 31412427 10.1002/mds.27802

[5] Bloem BR, Okun MS, Klein C. Parkinson’s disease. Lancet. 2021;397(10291):2284–2303. 33848468 10.1016/S0140-6736(21)00218-X

[6] Postuma RB, Berg D, Stern M, et al MDS clinical diagnostic criteria for Parkinson’s disease. Mov Disord. 2015;30(12):1591–1601. 26474316 10.1002/mds.26424

[7] Doty RL . Olfactory dysfunction in Parkinson disease. Nat Rev Neurol. 2012;8(6):329–339. 22584158 10.1038/nrneurol.2012.80

[8] Braak H, Del Tredici K, Rub U, de Vos RA, Jansen Steur EN, Braak E. Staging of brain pathology related to sporadic Parkinson’s disease. Neurobiol Aging. 2003;24(2):197–211. 12498954 10.1016/s0197-4580(02)00065-9

[9] Doty RL . Handbook of olfaction and gustation. 3rd ed. Wiley Blackwell; 2015.

[10] Doty RL, Shaman P, Kimmelman CP, Dann MS. University of Pennsylvania Smell Identification Test: A rapid quantitative olfactory function test for the clinic. Laryngoscope. 1984;94(2):176–178. 6694486 10.1288/00005537-198402000-00004

[11] Moessnang C, Frank G, Bogdahn U, Winkler J, Greenlee MW, Klucken J. Altered activation patterns within the olfactory network in Parkinson’s disease. Cereb Cortex. 2011;21(6):1246–1253. 21047984 10.1093/cercor/bhq202

[12] Hummel T, Fliessbach K, Abele M, et al Olfactory FMRI in patients with Parkinson’s disease. Front Integr Neurosci. 2010;4:125. 21120143 10.3389/fnint.2010.00125 PMC2991239

[13] Takeda A, Saito N, Baba T, et al Functional imaging studies of hyposmia in Parkinson’s disease. J Neurol Sci. 2010;289(1-2):36–39. 19720385 10.1016/j.jns.2009.08.041

[14] Westermann B, Wattendorf E, Schwerdtfeger U, et al Functional imaging of the cerebral olfactory system in patients with Parkinson’s disease. J Neurol Neurosurg Psychiatry. 2008;79(1):19–24. 17519323 10.1136/jnnp.2006.113860

[15] Tufo C, Poopalasundaram S, Dorrego-Rivas A, Ford MC, Graham A, Grubb MS. Development of the mammalian main olfactory bulb. Development. 2022;149(3):dev200210. 35147186 10.1242/dev.200210 PMC8918810

[16] Lu J, Wang X, Qing Z, et al Detectability and reproducibility of the olfactory fMRI signal under the influence of magnetic susceptibility artifacts in the primary olfactory cortex. Neuroimage. 2018;178:613–621. 29885483 10.1016/j.neuroimage.2018.06.008

[17] Wang J, Eslinger PJ, Doty RL, et al Olfactory deficit detected by fMRI in early Alzheimer’s disease. Brain Res. 2010;1357:184–194. 20709038 10.1016/j.brainres.2010.08.018 PMC3515873

[18] Yang QX, Dardzinski BJ, Li S, Eslinger PJ, Smith MB. Multi-gradient echo with susceptibility inhomogeneity compensation (MGESIC): Demonstration of fMRI in the olfactory cortex at 3.0 T. Magn Reson Med. 1997;37(3):331–335. 10.1002/mrm.1910370304 9055220

[19] Sobel N, Prabhakaran V, Desmond JE, et al Sniffing and smelling: Separate subsystems in the human olfactory cortex. Nature. 1998;392(6673):282–286. 9521322 10.1038/32654

[20] Zong X, Lee J, John Poplawsky A, Kim SG, Ye JC. Compressed sensing fMRI using gradient-recalled echo and EPI sequences. Neuroimage. 2014;92:312–321. 24495813 10.1016/j.neuroimage.2014.01.045 PMC4021580

[21] Sobel N, Prabhakaran V, Zhao Z, et al Time course of odorant-induced activation in the human primary olfactory cortex. J Neurophysiol. 2000;83(1):537–551. 10634894 10.1152/jn.2000.83.1.537

[22] Poellinger A, Thomas R, Lio P, et al Activation and habituation in olfaction—An fMRI study. Neuroimage. 2001;13(4):547–560. 11305885 10.1006/nimg.2000.0713

[23] Iravani B, Arshamian A, Ohla K, Wilson DA, Lundström JN. Non-invasive recording from the human olfactory bulb. Nat Commun. 2020;11(1):648. 32005822 10.1038/s41467-020-14520-9 PMC6994520

[24] Hua J, Qin Q, van Zijl PC, Pekar JJ, Jones CK. Whole-brain three-dimensional T2-weighted BOLD functional magnetic resonance imaging at 7 tesla. Magn Reson Med. 2014;72(6):1530–1540. 10.1002/mrm.25055 PMC4055555 24338901

[25] Miao X, Paez AG, Rajan S, et al Functional activities detected in the olfactory bulb and associated olfactory regions in the human brain using T2-prepared BOLD functional MRI at 7 T. Front Neurosci. 2021;15:723441. 10.3389/fnins.2021.723441 PMC8476065 34588949

[26] Miao X, Wu Y, Liu D, et al Whole-brain functional and diffusion tensor MRI in human participants with metallic orthodontic braces. Radiology. 2020;294(1):149–157. 31714192 10.1148/radiol.2019190070 PMC6939835

[27] Hua J, Miao X, Agarwal S, et al Language mapping using T2-prepared BOLD functional MRI in the presence of large susceptibility artifacts—Initial results in patients with brain tumor and epilepsy. Tomography. 2017;3(2):105–113. 28804779 10.18383/j.tom.2017.00006 PMC5552052

[28] Lundström JN, Gordon AR, Alden EC, Boesveldt S, Albrecht J. Methods for building an inexpensive computer-controlled olfactometer for temporally-precise experiments. Int J Psychophysiol. 2010;78(2):179–189. 20688109 10.1016/j.ijpsycho.2010.07.007 PMC2967213

[29] Doty RL, Brugger WE, Jurs PC, Orndorff MA, Snyder PJ, Lowry LD. Intranasal trigeminal stimulation from odorous volatiles: Psychometric responses from anosmic and normal humans. Physiol Behav. 1978;20(2):175–185. 662939 10.1016/0031-9384(78)90070-7

[30] Doty RL, Gregor T, Monroe C. Quantitative assessment of olfactory function in an industrial setting. J Occup Med. 1986;28:457–460. 3723219 10.1097/00043764-198606000-00015

[31] Doty RL, Shaman P, Dann M. Development of the University of Pennsylvania Smell Identification Test: A standardized microencapsulated test of olfactory function. Physiol Behav. 1984;32(3):489–502. 6463130 10.1016/0031-9384(84)90269-5

[32] Salimi-Khorshidi G, Douaud G, Beckmann CF, Glasser MF, Griffanti L, Smith SM. Automatic denoising of functional MRI data: Combining independent component analysis and hierarchical fusion of classifiers. Neuroimage. 2014;90:449–468. 24389422 10.1016/j.neuroimage.2013.11.046 PMC4019210

[33] Rolls ET, Huang C-C, Lin C-P, Feng J, Joliot M. Automated anatomical labelling atlas 3. Neuroimage. 2020;206:116189. 31521825 10.1016/j.neuroimage.2019.116189

[34] Castellan NJ . Nonparametric statistics for the behavioral sciences. McGraw-Hill; 1988.

[35] Lindquist MA, Loh JM, Atlas LY, Wager TD. Modeling the hemodynamic response function in fMRI: Efficiency, bias and mis-modeling. Neuroimage. 2009;45(1):S187–S198. 19084070 10.1016/j.neuroimage.2008.10.065 PMC3318970

[36] Zhao F, Wang X, Zariwala HA, et al fMRI study of olfaction in the olfactory bulb and high olfactory structures of rats: Insight into their roles in habituation. Neuroimage. 2016;127:445–455. 26522425 10.1016/j.neuroimage.2015.10.080

[37] Martin C, Grenier D, Thévenet M, et al fMRI visualization of transient activations in the rat olfactory bulb using short odor stimulations. Neuroimage. 2007;36(4):1288–1293. 17512755 10.1016/j.neuroimage.2007.04.029

[38] Poplawsky AJ, Kim S-G. Layer-dependent BOLD and CBV-weighted fMRI responses in the rat olfactory bulb. Neuroimage. 2014;91:237–251. 24418506 10.1016/j.neuroimage.2013.12.067 PMC3965612

[39] Schafer JR, Kida I, Rothman DL, Hyder F, Xu F. Adaptation in the rodent olfactory bulb measured by fMRI. Magn Reson Med. 2005;54(2):443–448. 16032685 10.1002/mrm.20588

[40] Xu F, Schaefer M, Kida I, et al Simultaneous activation of mouse main and accessory olfactory bulbs by odors or pheromones. J Comp Neurol. 2005;489(4):491–500. 16025460 10.1002/cne.20652

[41] Xu F, Kida I, Hyder F, Shulman RG. Assessment and discrimination of odor stimuli in rat olfactory bulb by dynamic functional MRI. Proc Natl Acad Sci U S A. 2000;97(19):10601–10606. 10973488 10.1073/pnas.180321397 PMC27071

[42] Li B, Gong L, Wu R, Li A, Xu F. Complex relationship between BOLD-fMRI and electrophysiological signals in different olfactory bulb layers. Neuroimage. 2014;95:29–38. 24675646 10.1016/j.neuroimage.2014.03.052

[43] Murphy MC, Poplawsky AJ, Vazquez AL, Chan KC, Kim SG, Fukuda M. Improved spatial accuracy of functional maps in the rat olfactory bulb using supervised machine learning approach. Neuroimage. 2016;137:1–8. 27236085 10.1016/j.neuroimage.2016.05.055 PMC4914461

[44] Muir ER, Biju KC, Cong L, et al Functional MRI of the mouse olfactory system. Neurosci Lett. 2019;704:57–61. 30951799 10.1016/j.neulet.2019.03.055 PMC11113078

[45] Xu F, Liu N, Kida I, Rothman DL, Hyder F, Shepherd GM. Odor maps of aldehydes and esters revealed by functional MRI in the glomerular layer of the mouse olfactory bulb. Proc Natl Acad Sci U S A. 2003;100(19):11029–11034. 12963819 10.1073/pnas.1832864100 PMC196921

[46] Zhao F, Wang X, Zariwala HA, et al fMRI study of the role of glutamate NMDA receptor in the olfactory adaptation in rats: Insights into cellular and molecular mechanisms of olfactory adaptation. Neuroimage. 2017;149:348–360. 28163142 10.1016/j.neuroimage.2017.01.068

[47] Cross DJ, Minoshima S, Anzai Y, et al Statistical mapping of functional olfactory connections of the rat brain in vivo. Neuroimage. 2004;23(4):1326–1335. 15589097 10.1016/j.neuroimage.2004.07.038

[48] Jia H, Pustovyy OM, Waggoner P, et al Functional MRI of the olfactory system in conscious dogs. PLoS One. 2014;9(1):e86362. 24466054 10.1371/journal.pone.0086362 PMC3900535

[49] Berns GS, Brooks AM, Spivak M. Scent of the familiar: An fMRI study of canine brain responses to familiar and unfamiliar human and dog odors. Behav Processes. 2015;110:37–46. 24607363 10.1016/j.beproc.2014.02.011

[50] Zhao F, Holahan MA, Houghton AK, et al Functional imaging of olfaction by CBV fMRI in monkeys: Insight into the role of olfactory bulb in habituation. Neuroimage. 2015;106:364–372. 25498426 10.1016/j.neuroimage.2014.12.001

[51] Boyett-Anderson JM, Lyons DM, Reiss AL, Schatzberg AF, Menon V. Functional brain imaging of olfactory processing in monkeys. Neuroimage. 2003;20(1):257–264. 14527586 10.1016/s1053-8119(03)00288-x

[52] Iravani B, Arshamian A, Schaefer M, Svenningsson P, Lundström JN. A non-invasive olfactory bulb measure dissociates Parkinson’s patients from healthy controls and discloses disease duration. NPJ Parkinsons Dis. 2021;7(1):75. 34408159 10.1038/s41531-021-00220-8 PMC8373926

[53] Doty RL, Kamath V. The influences of age on olfaction: A review. Front Psychol. 2014;5:72845. 10.3389/fpsyg.2014.00020 PMC3916729 24570664

[54] Tzeng W-Y, Figarella K, Garaschuk O. Olfactory impairment in men and mice related to aging and amyloid-induced pathology. Pflugers Arch. 2021;473:805–821. 33608800 10.1007/s00424-021-02527-0 PMC7895745

[55] Brumm MC, Pierz KA, Lafontant D-E, et al Updated percentiles for the University of Pennsylvania smell identification test in adults 50 years of age and older. Neurology. 2023;100(16):e1691–e1701. 36849448 10.1212/WNL.0000000000207077 PMC10115503

[56] Double KL, Rowe DB, Hayes M, et al Identifying the pattern of olfactory deficits in Parkinson disease using the brief smell identification test. Arch Neurol. 2003;60(4):545–549. 12707068 10.1001/archneur.60.4.545

