# Supplementary material for: Differential functional change in olfactory bulb and olfactory eloquent areas in Parkinson’s disease
Source: Brain Commun. 2024 Nov 16;6(6):fcae413. doi: 10.1093/braincomms/fcae413 (PMC11589462; doi:10.1093/braincomms/fcae413)
Supplement: fcae413_Supplementary_Data [file fcae413_supplementary_data.zip › Supplementary Table 1.docx]

**Supplementary Table 1. Comparison of functional MRI results in the olfactory bulb with and without spatial smoothing.**

| Olfactory bulb | Early PD patients  (n = 22) | Healthy controls  (n = 24) | *p* |
| --- | --- | --- | --- |
| *With spatial smoothing* |  |  |  |
| Activated voxel number | 28 ± 5^†^ | 32 ± 5 | 0.19 |
| ΔS/S (%) ^a^ from activated voxels in each subject | 1.94 ± 0.80 | 0.78 ± 0.43 | **0.03***^††^ |
| ΔS/S (%) from the combined activation map (30 voxels) | -0.03 ± 0.22 | -0.16 ± 0.17 | 0.15 |
| FWHM (s) of the HRF ^b^ | 2.31 ± 0.69 | 3.79 ± 0.29 | **0.01*** |
| TTP (s) of the HRF ^c^ | 4.80 ± 1.76 | 4.92 ± 0.81 | 0.40 |
| *Without spatial smoothing* |  |  |  |
| Activated voxel number | 27 ± 7 | 31 ± 5 | 0.33 |
| ΔS/S (%) from activated voxels in each subject | 1.95 ± 0.68 | 0.80 ± 0.55 | **0.03*** |
| ΔS/S (%) from the combined activation map (30 voxels) | -0.03 ± 0.24 | -0.17 ± 0.22 | 0.23 |
| FWHM (s) of the HRF | 2.33 ± 0.72 | 3.82 ± 0.44 | **0.02*** |
| TTP (s) of the HRF | 4.56 ± 1.83 | 4.77 ± 1.02 | 0.32 |

^†^ Mean ± Standard error

^††^ Bold values indicate statistically significant results.

^a^ Olfactory functional MRI signal change: ΔS/S = 100 × (stimulus-on signals - stimulus-off signals) / stimulus-off signals %.

^b^ FWHM: full width at half maximum (s) estimated from the hemodynamic response function (HRF).

^c^ TTP: time to peak (s) estimated from the hemodynamic response function (HRF).
